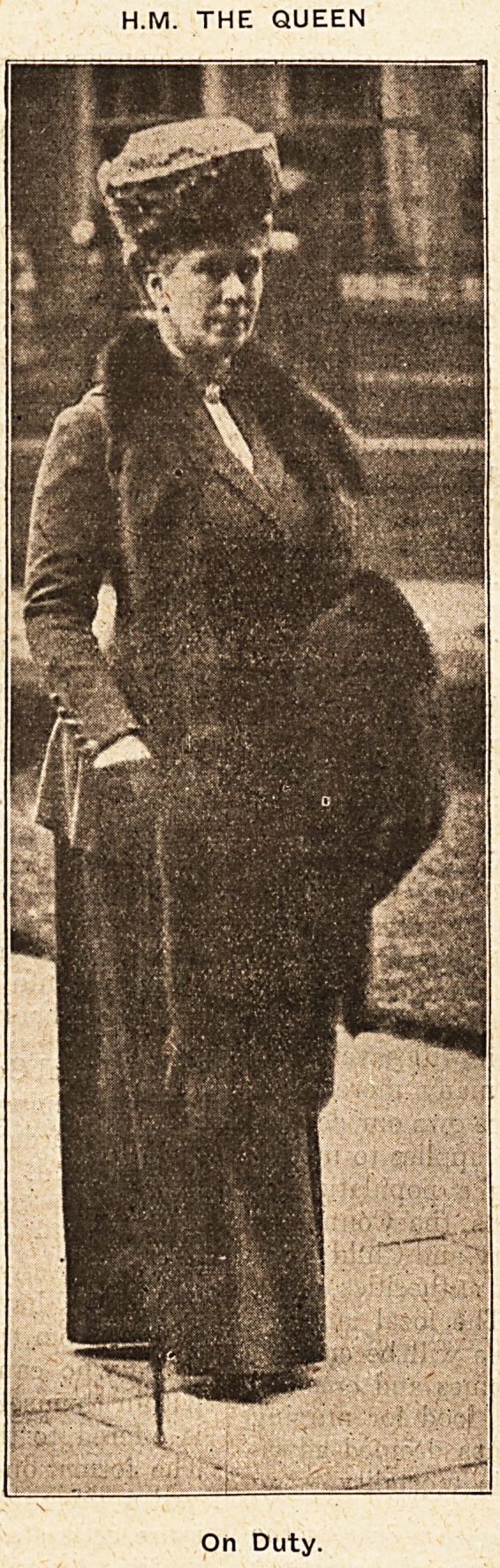# Round the Hospitals

**Published:** 1918-04-20

**Authors:** 


### ROUND THE HOSPITALS.

We are indebted to the *American Journal of Nursing* for the announcement that Miss Annie W. Goodrich has been called to service in the war. She is the President of the American Nurses' Association, and assistant professor of the Department of Nursing and Health, Teachers' College, and has been promoted to an appointment under the War Department as Chief Inspecting Nurse of the U.S. Army hospitals at home and abroad. The American journal declares with justice: "Miss Goodrich is peculiarly fitted to fill this position because of her varied experience in hospitals and as Inspector of Training-Schools in New York State; and the profession may feel proud that the Government has made so wise a selection." Miss Goodrich is entitled to, and will undoubtedly receive, a warm welcome in Great Britain and from the fighting forces at the Front. She has a wide and special knowledge of the rise and progress of nursing, and of nurse-training in the United States, and takes a warm interest in British nursing. She has had notable success in connection with the responsible work she has undertaken for the development of trained nurses, their education and training. She is a thoughtful, able woman, with an attractive personality. Everybody on this side who has the pleasure of meeting her will find her to be full of sound knowledge, and that she takes an independent and far-reaching interest in everything affecting the advancement and development of the higher standards of nursing everywhere. The Inspector of Training-Schools in New York State, Miss Elizabeth C. Burgess, has been selected by Miss Goodrich as her assistant. There will be plenty of work for both of them to do. We wish for them unbroken health and the most inspiriting experiences in their military career.

The admission of women to the suffrage has already been attended by one gratifying result, on which we congratulate all concerned. The National League for Opposing Woman Suffrage, at a special council, has decided unanimously to dissolve the league. A large number of delegates from various parts of the country were present, and it has been decided to devote the surplus funds, which amount to some thousands of pounds, to the Royal National Pension Fund for Nurses, to be specially applied, as far as possible, during the war to the Queen Alexandra Relief Fund for War Nurses. This encouraging announcement will no doubt be noted by everyone interested in the successful establishment of the College of Nursing on a sound financial basis, because it inferentially proves how warm and widespread is the appreciation throughout the country of this wonderful success attaching to the efforts of nurses to help themselves. The Royal National Pension Fund has become the greatest thrift agency amongst women workers in the world, and we look forward to the time when the College of Nursing will develop into the Educational Centre for the training of nurses and the development of the highest standards of nursing to which every nurse, from all parts of the Empire, will turn. The progress it has already made, and the ever-increasing number of nurses who are registering with the College, are notable facts. They should hasten the growth of its Endowment Fund and secure the united support of all independent people, who appreciate the services our nurses have rendered, and regard it a privilege to extend to them every possible encouragement and help in their power.

The glorious deeds of the Army in Flanders and France continue to stir the whole Empire with pride. To emulate their steadfastness in humble degree is the only comfort left for the women. Our sisters and nurses have given splendid testimony during the recent rearguard actions of their ability to do this. Immense relief was afforded by the news which has now come through that no officer, sister, or man of the clearing-station staff has been lost, and that no single patient, who had once been able to reach these units, has been left behind with the enemy. The *British Medical Journal* states that the more advanced clearing-stations were moved back at once without difficulty, but very soon the other medical units were threatened under the rapid advance of the enemy, and the difficulty of removing the wounded became increasingly great. One unit after another had to be evacuated, sometimes within forty-eight or even twenty-four hours of being opened up, and at each move some of its equipment was lost. "Occasionally some of the last cases were got away whilst the infantry were preparing to make a stand in the clearing-station grounds, with the field-guns firing over their heads from new positions behind." All that could be done was to dress and feed the patients and perform a few urgent operations.

The imminent peril to which the nursing sisters were exposed in common with their *confrères* at the clearing-stations found them imperturbable. Their gallantry and oblivion of danger was never in question, and was evidenced in many striking ways.

**ROUND THE HOSPITALS—(*continued*).**

"One group of nurses, we learn, stood quietly beside the railway line whilst their station was being shelled, waiting for a train which might or might not succeed in reaching them; yet next day they were at work in a new place as though nothing had happened." Let us hope that the names of those who took part in this historic retreat will be permanently recorded. It has been announced that a considerable number of casualties occurred among the nursing *personnel*, and the list is anxiously awaited. One American nurse attached to a "surgical team," lent by a Philadelphia hospital, was wounded.

QUEEN ALEXANDRA has intimated how greatly she was impressed with the excellence of the arrangements made in connection with the Memorial Service for Nurses who have fallen in the war, and has conveyed her warm appreciation to the Rev. A. Lombardini, who so successfully organised the service. The collection in St. Paul's amounted to £118 9s., and the sale of the Forms of Service realised £30. The proceeds will be devoted to the war work of the British Red Cross Society and the Order of St. John of Jerusalem.

THE extent to which the art of nursing has aided in uniting women from all over the world in a common aim was never more strikingly illustrated than in St. Paul's on the 10th inst. The foremost seats on the south side of the Dome were accorded to the Allied nursing contingents, numbering many American nurses in their dark-blue uniforms, whose collaboration was never more imperatively needed than at this moment. Side by side with them were the Canadians and a goodly muster of nurses from South Africa. On the north side were grouped members of the Queen Alexandra's Imperial Military and Naval Services and Reserves, with members of the Territorial Nursing Force. The seats in front of these large and representative bodies were occupied by sisters and nurses from the Australian and New Zealand units, to whom precedence was given out of a desire to emphasise the value of the generous nursing reinforcements sent over from the Dominions. Hundreds of civilian matrons, sisters, and nurses were present, including many members of the Jubilee Institute. It was a striking testimony to the numbers and influential character of the Empire Nursing Service that the gathering numbered little short of 5,000 in this time of extreme nursing pressure, when the military necessities are making such unprecedented demands.

H.M. THE QUEEN

On Duty.

A NEW Army Council instruction has been issued which defines more exactly the amount of leave which can be granted to members of the Queen Alexandra's Imperial Military Nursing Service and Reserve, the Territorial Force Nursing Service and Reserve, assistant nurses, and members of the Voluntary Aid Detachments. The maximum leave, which the instruction states should be given "whenever possible," is as follows:—Matrons, forty-two days; sisters, thirty-five days; staff nurses, twenty-eight days; assistant nurses, twenty-three days. V.A.D. members and military probationers on continuous service are to get seven days during the first six months, fourteen days during the second and every subsequent six months. Should the necessities of the troops permit this scale to be maintained there would be far less wastage than there is at present among the valuable material made up of nurses' energies. But a crisis such as that which has developed in France and Flanders must inevitably drive a coach-and-four through the most admirable regulations. Sir Douglas Haig's pathetic reminder, "We are all tired," finds an echo in every nurse's heart.

THE authorities have dealt very promptly with the injurious statements publicly circulated about the Women's Auxiliary Army Corps, and have been able triumphantly to vindicate this fine body of women from the scandals invented about them. It has not been difficult to prove that discipline has been well maintained, for the close supervision rendered necessary by war conditions makes concealment of wrongdoing impossible. We congratu-

ROUND THE HOSPITALS—(*continued*).

late Mrs. Burleigh Leach on the able manner in which the vague but no less on that account injurious slanders have been met and refuted. They have been circulated by precisely the same kind of people who make an outcry one day about the rules of discipline maintained among Army nurses and the next invent complaints or distort harmless incidents to their discredit or that of their chiefs. The march of the W.A.A.C.s to St. Paul's to-day will give Londoners an opportunity of judging their quality, and it has been finely conceived to unite them in prayer and thanksgiving at the national Cathedral.

In going over the nurses' quarters in a well-known London hospital recently our attention was called to the fact that black American cloth had been substituted for white table-cloths on the tables set for the evening meal. We learned that this was a war economy, cheerfully agreed to by the nurses, which had been in use for the last two years and had resulted in a decided diminution of the washing bill. The black effect, with flower decorations, is said to be quite good, and we can imagine that meals taste just as well without their usual livery. The principle, however, is not one for partial application. If one section of the household loses its white table-cloths, all should do so. Economies of this type are only objectionable when they suggest that the training-school for nurses may be skimped of luxuries still deemed indispensable for other sections of the institution household.

The new Maternity and Child Welfare Bill introduced by Mr. Hayes Fisher will undoubtedly be pressed through by the Government, notwithstanding the adverse criticism which has been lavished upon it in certain quarters. It is a singularly brief and uncontentious measure, but it is claimed on its behalf by those who know that it will save 1,000 babies a week from death and many more from that slow devitalisation to which we owe our deplorable tale of incurable children. It applies to metropolitan or urban districts having a population of over 20,000, and in these districts the Council is empowered to establish a Maternity and Child Welfare Committee, on which expert authorities, male and female, may be co-opted. The local authorities acting through this Committee will be enabled to provide as required lying-in homes and *crèches*, to provide pure milk for infants, food for nursing mothers, and promote any schemes deemed advisable for the prevention of infantile mortality.

In this connection we would like to point out the advisability of recognising the matrons of the county hospital or leading Poor-Law infirmary as fit persons to sit on a Child Welfare Committee. We have been often struck by the extent to which matrons are ignored by the Borough and County Councils within whose sphere their work is done. Few persons have better opportunities than they of forming a correct judgment on matters affecting child welfare. Most of the sick children in the borough pass through their hands, and their influence with the mothers is authoritative and irresistible. Yet it does not seem to occur to local magnates that this rich mine of information and practical experience lies wholly unused. Any restless crank or well-disposed but ignorant amateur or newly qualified health visitor is preferred before the one woman in the town who really knows. All this ought to be altered, and matrons should be summoned to the exercise of their undoubted powers in matters affecting the health of the community.

The London County Council has organised a series of lectures, primarily for the benefit of their own domestic science teachers, but open to others, and they are of a nature to be particularly advantageous to the second- and third-year probationers in Poor-Law and other institutions, where sometimes a lack of variety may be experienced in the curriculum. The lectures are given at the London Day Training College, Southampton Row, at 6.30 on Tuesday evenings. Tickets for the course, 7s., may be obtained from the Education Officer, L.C.C. Offices, Victoria Embankment. On April 23 Mr. John Wilson lectures on "Scientific Method in Relation to Housewives' Problems, Cooking, Laundry, and Housework"; on April 30, Mr. Cyril Burt on "Child Training"; on May 7, Miss Punnett on "Domestic Calculations"; on May 14, Mr. R. Weir on "House Planning in Relation to Housing Problems"; on May 28, Miss Buck on "The Needle Subjects." Few Boards of Guardians or house committees which have learned to take an intelligent interest in the education of their nurses would grudge the few shillings' expenditure needed to enable some of their probationers to profit from such a course. To utilise opportunities of this kind, which not infrequently occur in London, would let fresh air into many a stale, and therefore slack, training-school and tend to stimulate zeal.

The Local Government Board is taking the first step towards wiping out a very heavy national reproach in giving orders for the compilation of a Register of the Blind. Few Poor-Law infirmaries are without one or two blind men and women whose lot is peculiarly animal and monotonous in the entire absence of any occupation or means of recreation. The blind have usually a very lively mentality. Cut off from the little play of interests which divert those who can watch their neighbours they retire within themselves and suffer acutely when no food is offered to their minds by way of work or play. The formation of a complete list of these sensitive and much neglected members of society will enable some estimate to be formed of the right manner in which to ameliorate their lot. It is by no means an easy thing to help blind people who have no relatives to look after them, but it is liberty they crave for even more passionately than do their fellow prisoners in the infirm wards of the workhouse. The problem of the blind needs a constructive policy framed with knowledge and sympathy, and both these are abundantly at the disposition of the authorities whenever they may desire to lay hands on them.

☞ For "An Imperial Union of Nurses," see p. 56; Essex County Hospital, Important Letters, see p. 53; Matrons' and Sisters' Appointments, see p. 64.

## Figures and Tables

**Figure f1:**